# Food Allergens and Essential Oils in Moisturizers Marketed for Children in Japan

**DOI:** 10.7759/cureus.34918

**Published:** 2023-02-13

**Authors:** Kenta Horimukai, Misako Kinoshita, Yasutoshi Shamoto, Takeshi Inoue, Hisashi Tanida

**Affiliations:** 1 Department of Pediatrics, Jikei University Katsushika Medical Center, Tokyo, JPN; 2 Department of Pediatrics, Holy Spirit Hospital, Nagoya, JPN; 3 Department of Pediatrics, Toyohashi Municipal Hospital, Toyohashi, JPN

**Keywords:** volatile, oils, nuts, fruit, cosmetics, allergens

## Abstract

Introduction

Personal skincare leave-on products increase the risk of food allergies. Parents must be imparted with an elevated degree of cognizance regarding the allergenic nature of pediatric skincare products.

Material and methods

We aimed to examine the data inferred from the promotional material on labeling these products about their proclivity to elicit skin sensitization. This study investigated the relationship between food allergens and essential oil ingredients and highlighted marketing terms, product prices, and ratings of moisturizers for children that are sold on Amazon, Japan. We searched and recorded the product labels and website marketing terms, price (per gram or milliliter), the number of reviews, and allergens and investigated the relationship between the percentage of food allergens in those products and marketing terms, price, and the number of Amazon reviews.

Results

Among the 164 pediatric skincare products we included, 144 (87.8%) that were manufactured in Japan were the most common; 7 (4.3%), 15 (9.1%), 23 (14.0%), 24 (14.6%), and 54 (32.9%) contained the eight regulated food allergens, grain, nut, fruit, and essential oils, respectively. Marketing terms emphasizing “natural/organic” were more likely to contain grain allergens and essential oils and were more expensive with and without “organic” labeling, respectively, whereas those labeled with marketing terms emphasizing “hypoallergenic” were less likely to contain fruit allergens or essential oils. Products with fewer Amazon reviews were more likely to use the marketing term “natural/organic” and had a higher grain allergen content.

Conclusion

In Japan, 4.3% of children's skincare products sold on Amazon contain eight food allergens that should obligatorily be labeled when included in food products. In addition, more than 10% of these children's skin care products contain ingredients derived from nuts, while more than 30% contain fruit extracts or essential oils.

## Introduction

Worldwide, the number of children with food allergies is increasing, and 8% of children in the USA and 5% of young children in Japan have food allergies [1,2]. In a single Japanese urban center, the incidence of food allergies has been demonstrated to escalate annually [3]. Childhood food allergy decreases family quality of life and confers nutritional and financial burdens [4]. Therefore, avoiding risk factors for food allergies is essential. Furthermore, food allergens, such as peanuts and oats, are risk factors for food allergies even on skin contact [5,6]. Essential oils derived from fruits and plants can induce sensitization following skin contact [7,8]. As advanced by the Dual Allergen Exposure Hypothesis, it is widely acknowledged that topical exposure to food allergens increases the susceptibility of individuals to food allergies.

Many personal care products for children contain food ingredients, although the mechanism of percutaneous sensitization increases the risk of food allergies [5,6]. Moreover, various marketing terms suggest that these food ingredients have beneficial effects on the skin, and products purchased on e-commerce sites are more valued by consumers, thereby reinforcing the purchasing behavior of consumers [9]. Thus, the consumers’ emphasis on marketing terms and e-commerce site reviews may constitute risk factors for selecting personal care products that pose a risk of allergen sensitization.

We analyzed the relationship between food allergens and essential oil content and emphasized marketing terms used for personal care products for children sold on Amazon, one of the largest e-commerce sites in Japan, and product prices and ratings. We investigated the impact of these factors on the content of allergens and essential oils in personal skin care products for children. We believe that the examination of the correlation between allergens and essential oil elements in children's skin care products and their advertising branding would furnish crucial information for parents in the act of making informed buying choices for such items.

## Materials and methods

On June 27 and 28, 2022, we searched for products on Amazon using the terms “moisturizer,” “baby,” and “children,” which were separated by one-byte spaces. After recording all products displayed, we clicked “Next” and checked all products on all pages. This study was exempted from the need for ethical review because it is not a survey of human subjects.

The web browser used for the search was Google Chrome (version: 102.0.5005.115, 64-bit), and incognito browsing was used to avoid the effects of personalized data retrieval. Product label and website marketing terms, price (per gram or milliliter), and the number of reviews and ratings were recorded.

In Japan, the "Code of Fair Competition for the Labeling of Cosmetics" sets forth guidelines for general consumers to make informed selections of products [10]. However, there are no strict regulations for “names by type,” which are descriptions of names that describe uses and names that describe dosage forms.

We defined 10 products ascertained to be personal-care skin-moisturizing products for children: “cream,” “ointment,” “body butter,” “body balm,” “oil,” “lotion,” “gel,” “powder,” “spray,” and “foam.” Additionally, we categorized and summarized marketing terms believed to have the same meaning. The marketing terms we searched for and their possible suggested meanings are listed in Table 1.

**Table 1:** Classification of marketing terms based on product packaging and product web pages

Marketing terms	Themes that marketing terms may emphasize
Contains no additives	Emphasize that the product has few additives
Contains no fragrance	
Contains no coloring matter	
Minimal skin irritation	Emphasize that the product is less irritating to the skin
Low allergenicity	
Suitable for sensitive skin	
Organic	Emphasize that the product is made with natural and organic ingredients
Natural	
Nature	
Allergy testing is in place	Emphasize that the product is less allergenic
Skin irritation tests are performed	

We excluded products that manifest in Amazon's search results but are no longer in production and inaccessible during the moment of search, products not labeled for children, and products intended to treat specific conditions (e.g., sweat rash). The manufacturer’s website and product photos were used to obtain information on raw materials. However, in cases in which the raw materials in the product could not be determined after searching for sufficient information, the corresponding product was purchased, and the ingredients were confirmed.

The number of ratings and rating scores were recorded based on Amazon reviews. Products with zero ratings were excluded from statistical evaluation regarding Amazon reviews. Additionally, we examined whether the number of Amazon reviews was associated with food allergen content and marketing terms. We categorized the products into groups based on the number of reviews and compared the top quartile of products with the highest Amazon review counts to those with the lowest Amazon review count to examine the relationship between organic labeling and review frequency, to compare the relationship between organic labeling and the number of reviews.

In the selected personal-care skin-moisturizing products, we identified eight food allergens regulated by Japanese food allergen-labeling requirements. Representative grain, fruit, and essential oil allergens were selected, and this information was entered into a Microsoft Excel for Microsoft 365 (64-bit) MSO Version 2207 (16.0.15427.20182) (Microsoft Corp, Redmond, WA, USA) spreadsheet (Table 2). Fisher’s exact test was used to determine the differences in continuous variables, and values are presented as means and SD. Differences in categorical variables were evaluated with the chi-square test, and P-values lower than 0.05 were considered significant. Analyses were performed using IBM SPSS Statistics for Windows, Version 17 (Released 2008; IBM Corp., Armonk, New York, United States).

**Table 2:** Food allergens studied in this study

Classification of food allergens	Food allergens
Eight food allergens subject to Japan's food allergen labeling requirements	Hens egg
	Cow's milk
	Fish
	Shrimp
	Crab
	Buckwheat
	Walnut tree
	Peanuts
Grain allergens	Wheat
	Rice
	Coix seeds (*Coix lacryma-jobi var. ma-yuen*)
	Oats (*Avena sativa*）
Nut allergens	Walnut tree
	Almond
	Macadamia nut
	Sesame (seeds)
Fruit allergens (includes pulp and seeds)	Avocado (*Persea americana*)
	Mandarin orange/Orange
	Lemon
	Grape
	Kiwi
Essential oils	Lavender
	Rosemary (*Salvia rosmarinus*)
	Narrow-leaved paperbark tea tree (*Melaleuca alternifolia*)
	Rose geranium (*Geranium rosacea*)
	Thymus vulgaris
	Rose hip
	Common sage (*Salvia officinalis*)
	Aloe vera
	Calendula officinalis
	Chamomile (*Matricaria recutita*)

## Results

During the initial search, 164 pediatric personal-care skin products met the criteria, which were defined to include leave-on skincare products. Products manufactured in Japan accounted for the largest share (n=144 products, 87.8%), whereas 20 products were manufactured outside Japan (five products (3.0%) in Malaysia, three (1.8%) in the People’s Republic of China, two (1.2%) in Thailand, two (1.2%) in Germany, one (0.6%) in New Zealand, one (0.6%) in Italy, one (0.6%) in Switzerland, one (0.6%) in France, and four [2.4%) with unknown country of origin).

Lotions were the most common product type and included 59 products (36.0%), followed by cream (n=51 products; 31.1%) and gel (n=34 products; 20.7%) (Table 3).

**Table 3:** Classification of children's skincare products

Skincare products	Number of products
	n (%)
Lotion	59 (36.0)
Cream	51 (31.1)
Gel	34 (20.7)
Oil	9 (5.5)
Body butter	5 (3.0)
Spray	3 (1.8)
Ointment	1 (0.6)
Body balm	1 (0.6)
Foam	1 (0.6)
Powder	0 (0.0)

Seven products (4.3%) contained the eight food allergens regulated by the Japanese food allergen labeling requirements, 15 (9.1%) contained grain allergens, 23 (14.0%) contained nut allergens, 24 (14.6%) contained fruit allergens, and 54 (32.9%) contained essential oils (Table 4). The disparity in the final count is a result of the inclusion of numerous allergens within a single topical cosmetic preparation. Specifically, Macadamia nuts (10.4%), oranges (9.8%), and rice (6.7%) were the more common ingredients. Additionally, various essential oil components, including lavender (14.6%), rosemary (12.2%), and aloe vera (12.2%), were present.

**Table 4:** Food allergens studied in this study

Classification of food allergens	Food allergens	Number of products containing allergens (%)	Total (%)
		n (%)	n (%)
Eight food allergens subject to Japan's food allergen labeling requirements	Hens egg	1 (0.6)	7 (4.3)*
	Cow's milk	6 (3.7)	
	Fish	1 (0.6)	
	Shrimp	0 (0.0)	
	Crab	0 (0.0)	
	Buckwheat	0 (0.0)	
	Walnut tree	0 (0.0)	
	Peanuts	0 (0.0)	
Grain allergens	Wheat	0 (0.0)	15 (9.1)*
	Rice	11 (6.7)	
	Coix seeds (*Coix lacryma-jobi var. ma-yuen*)	5 (3.0)	
	Oats(*Avena sativa*）	0 (0.0)	
Nut allergens	Walnut tree	0 (0.0)	23 (14.0)*
	Almond	4 (2.4)	
	Macadamia nut	17 (10.4)	
	Sesame (seeds)	4 (2.4)	
Fruit allergens (includes pulp and seeds)	Avocado (*Persea americana*)	2 (1.2)	24 (14.6)*
	(Mandarin) Orange	16 (9.8)	
	Lemon	4 (2.4)	
	Grape	4 (2.4)	
	Kiwi	0 (0.0)	
Essential oil	Lavender	24 (14.6)	54 (32.9)*
	Rosemary (*Salvia rosmarinus*)	20 (12.2)	
	Narrow-leaved paperbark tea tree (*Melaleuca alternifolia*)	3 (1.8)	
	Rose geranium (*Geranium rosacea*)	1 (0.6)	
	Thymus vulgaris	8 (4.9)	
	Rose hip	11 (6.7)	
	Common Sage (*Salvia officinalis*)	9 (5.5)	
	Aloe vera	20 (12.2)	
	Calendula officinalis	11 (6.7)	
	Chamomile (*Matricaria recutita*)	25 (15.2)	
*The disparity in the final count is a result of the inclusion of numerous allergens within a single topical cosmetic preparation			

The average product price (per gram or per milliliter) was 19.19 ± 32.7 yen (range 1.3-353.6 yen), the number of reviews on Amazon was 277.1 ± 502.8 (range 0-3602), and the rating was 4.26 ± 0.326 (range 2.0-5.0). Table 5 shows the frequency of products labeled with marketing terms defined as variables: 141 products (86.0%) emphasized low additives, 114 (69.5%) emphasized mildly irritating character, 73 (44.5%) emphasized natural/organic, and 76 (46.3%) emphasized hypoallergenic nature. Furthermore, we investigated the association between marketing terms in these four categories and products containing allergens (Table 6).

**Table 5:** Themes that marketing terms may emphasize

Marketing terms	Number of products (%)
Emphasizes that the product is low in additives	141 (86.0)
Emphasizes that the product is less irritating to the skin	114 (69.5)
Emphasizes that the product is made with natural and organic ingredients	73 (44.5)
Emphasizes that the product is less allergenic	76 (46.3)

**Table 6:** Relevance of allergen content in skin care products and marketing terms

Emphasized marketing terms	Eight food allergens subject to Japan's food allergen labeling requirements			Grain allergens			Nut allergens			Fruit allergens			Essential oil		
	Contain group (n=7)	Not contain group (n=157)	p-value	Contain group (n=15)	Not contain group (n=149)	p-value	Contains group (n=23)	Not contain group (n=141)	p-value	Contain group (n=24)	Not contain group (n=140)	p-value	Contains group (n=54)	Not contain group (n=110)	p-value
Emphasizes that the product is low in additives	5/7 (71.4%)	136/157 (86.6%)	0.225	11/15 (73.3%)	130/149 (87.2%)	0.231	21/23 (91.3%)	120/141 (85.1%)	0.745	20/24 (83.3%)	121/140 (86.4%)	0.750	47/54 (87.0%)	94/110 (85.5%)	1.0
Emphasizes that the product is less irritating to the skin	5/7 (71.4%)	109/157 (67.5%)	1.0	10/15 (66.7%)	104/149 (69.8%)	0.775	13/23 (56.5%)	101/141 (71.6%)	0.151	16/24 (66.7%)	98/140 (70.0%)	0.811	34/54 (63.0%)	80/110 (72.7%)	0.212
Emphasizes that the product is made with natural and organic ingredients	2/7 (28.6%)	71/157 (45.2%)	0.463	11/15 (73.3%)	62/149 (41.6%)	<0.05	14/23 (60.9%)	59/141 (41.8%)	0.114	15/24 (62.5%)	58/140 (41.4%)	0.075	36/54 (66.7%)	37/110 (33.6%)	<0.01
Emphasizes that the product is less allergenic	2/7 (28.6%)	74/157 (47.1%)	0.452	6/15 (40.0%)	70/149 (47.0%)	0.787	11/23 (47.8%)	65/141 (46.1%)	1.00	6/24 (25.0%)	70/140 (50.0%)	<0.05	16/54 (29.6%)	60/110 (54.5%)	<0.01

Products with marketing terms emphasizing “natural and organic” were more likely to contain grain allergens and essential oils and had a higher cost per milliliter (gram; 26.8 ± 45.4 and 13.1 ± 14.0 yen for the groups with and without the term “organic,” respectively, in the labeling). In contrast, products with the marketing term emphasizing “hypoallergenic” were less likely to contain fruit allergens or essential oils. Additionally, the number of Amazon reviews was significantly associated with “organic” in the labeling and the presence of grain allergen content.

We subdivided the products into groups according to the number of reviews and compared those having 25% or more Amazon review counts (Group A ≥306 Amazon reviews) with those having 25% or fewer Amazon reviews (Group B ≤28 Amazon reviews). The group with fewer Amazon reviews had more organic labeling (16 of 40 products in Group A vs. 26 of 40 products in Group B; p=0.043) and higher grain allergen content (1 of 40 products in Group A vs. 8 of 40 products in Group B; p=0.029).

## Discussion

The risk of percutaneous sensitization from personal-care products became widely recognized after Lack et al. reported, in 2003, that children who used peanut-containing personal care products during infancy developed a high rate of peanut allergy [5]. This generated considerable attention to food allergens in over-the-counter skin care products meant to be left on the skin. However, few reports have examined the extent to which food allergens and essential oils are present in moisturizing personal-care products to be left on children’s skin. To our knowledge, only one report has examined food allergens in children’s skin care products [11]. The authors stated that the most common food allergens in personal skin care products for children are almonds, wheat, soy, oats, and sesame. However, our study found that macadamia nuts, oranges, rice, and various essential oil components were more common ingredients in skin care products for children in Japan.

In Japan, nuts and fruits are common causes of new food allergies after the age of 1 year, and the prevalence of egg, milk, nut, and wheat allergies is substantial [12]. Furthermore, the prevalence of nut allergy in Japan is increasing [2,13]. For example, owing to the increased prevalence of walnut allergy, walnuts have been added to Japan’s list of food allergen-labeling control foods in 2022 [13]. In Japan, these foods are not to be started early in complementary diets. Accordingly, proceeding with transdermal sensitization by these products before the complementary diet is initiated would not be beneficial.

Adomaite et al. showed significantly more food allergens in personal care products for the skin when marketing terms emphasizing “natural and organic” were presented, that is, almonds and wheat were the most common allergens [11]. This may be because the impression that products labeled organic are safe as skin care products for children is widely accepted by caregivers and more likely to be chosen as a marketing term [14]. However, our research showed that when marketing terms emphasizing “natural and organic” were included, grains and essential oils were often included. Therefore, the types of food allergens in skin care products may vary by country or region, even if the same marketing terms are used. In contrast, products with marketing terms that emphasized “low allergenicity” had low fruit and essential oil content. Accordingly, we inferred that these marketing terms were labeled with consideration for fruits and essential oils, and not for nuts and grains.

The prevalence of food allergies to hens’ eggs, milk, wheat, and nuts is high in Japan [12]. However, in this study, small amounts of these food allergens were present in children’s skin cosmetics. A well-known wheat-allergic patient developed wheat allergy owing to percutaneous sensitization to soap-containing wheat in Japan [15]. The majority of products in this study were manufactured in Japan. Therefore, they may have avoided the inclusion of well-known food allergens in their skin care products. However, the content of nuts, grains, fruits, and essential oils is not minimal.

Many consumers will refer to product prices and Amazon reviews when selecting products, and these factors were examined in this study. Products labeled with the marketing term “natural and organic” were more expensive and more likely to contain grain-based allergens and essential oils. Moreover, products with more Amazon reviews had less “organic” labeling and less grain allergen content. Thus, products with fewer reviews may have structured their marketing strategy by emphasizing the “natural and organic” label, which was associated with a higher food allergen content. Therefore, there is a requirement for conscientiousness regarding the marketing vocabulary.

Conversely, topically applied food ingredients and essential oils may confer skin-beneficial effects. Some ingredients, such as oats, calendula, and aloe vera, are effective in treating diaper dermatitis and other skin problems of infancy [16,17]. However, oats cause sensitization [6], and calendula cross-reacts in individuals with sensitization to Asteraceae pollen [18]. The increasing rate of pollen sensitization in Japanese children necessitates caution concerning these ingredients [19].

A limitation of this study is its cross-sectional design, which precludes the determination of a causal relationship between personal skin care products that can act as food allergen-sensitizers and cause food allergies. A limitation of this study is the reliance on raw material and product labeling verification based on information obtained from the websites. Thus, the possibility of drawing definitive conclusions is precluded. However, previous studies have shown that these food allergens and essential oils can cause sensitization. Therefore, it is important to determine the extent to which food allergens and essential oils are present in children’s skin care products. Furthermore, it is possible that not all food allergens were identified from the product labels. However, in this study, the researcher visually verified all labels; thus, it is unlikely that any allergens were overlooked. Another limitation is that the only e-commerce site searched was Amazon. However, Amazon is one of the largest e-commerce sites in Japan, and we believe that it is possible to identify most products sold in Japan and trends in personal skin care products.

## Conclusions

In conclusion, food allergens and essential oil ingredients in personal skin care products for children sold in Japan were investigated, and more than 10% contained nuts, grains, and fruits, whereas more than 30% of the products contained essential oils. Marketing terms that emphasized “natural and organic” were associated with products with an increased percentage of grain and essential oil content, whereas terms emphasizing “hypoallergenic” were associated with products with lower fruit and essential oil content. Furthermore, the risk of products containing food allergens might be lower for products with more Amazon reviews and lower prices. The results of this study may help caregivers select personal skin care products for their children. It is imperative for parents to exercise vigilance when procuring skin care products for their offspring, as some of these products may pose the possibility of eliciting an adverse sensitization response. In addition, it is advisable for them to request that the purveyors of these products update the accompanying user guide labeling to ensure informed choices for future acquisitions.
